# Should Human Immunodeficiency Virus Testing Be Replaced With Combined Blood-Borne Virus Testing?

**DOI:** 10.1093/ofid/ofad668

**Published:** 2024-01-11

**Authors:** Chloe Orkin

**Affiliations:** SHARE Collaborative, Blizard Institute, Queen Mary University of London, London, UK; Barts Health NHS Trust, London, UK

**Keywords:** HIV testing, Blood-borne virus testing, BBV testing, emergency department

## Abstract

Standalone HIV testing is a missed opportunity- a recent systematic review showed eight cases of viral hepatitis would be missed for each new HIV diagnosis. The review builds on a decade of research demonstrating the additional benefit of BBV testing.

Globally, more than 1.1 million individuals die from hepatitis B virus (HBV) and hepatitis C virus (HCV) each year; however, only 10.5% of individuals with HBV and 21% of individuals with HCV are diagnosed [1–3]. This is in stark contrast to human immunodeficiency virus (HIV), where 85% of individuals with HIV are diagnosed [4]. Seventy-five percent of adults with HIV are on treatment, which compares very starkly with only 2.3% of those with HBV and 13% with HCV [1–4]. In 2023 the World Health Organization (WHO) pledged to eliminate the HIV, HBV, and HCV epidemics by 2030 and aligned with the United Nations General Assembly sustainable development goals [5, 6]. These important targets are yet to be achieved, which is particularly unfortunate given that HCV can be cured with antivirals for $60/year [2, 7]. HBV is treatable with antivirals costing just $29/year [1, 3].

In this issue of *Open Forum Infectious Diseases*, Beard and Hill present the findings of an impressive systematic review of 175 studies sampling >14 million individuals. They compare the efficacy and costs of offering triple test programs against stand-alone HIV programs to support the WHO guidelines [8]. Their findings are both clear and striking.

The authors evaluated the prevalence of HIV, HBV, and HCV across 9 different population groups: general population, hospital attendees, blood donors, people who inject drugs, people experiencing homelessness, people seeking asylum, people who are incarcerated, pregnant people, and gay and bisexual men who have sex with men (GBMSM). Apart from in GBMSM, across the 9 populations they evaluated, the combined prevalence of HBV and HCV is greater than that of HIV. By delivering combined blood-borne virus (BBV) testing, for each case of HIV identified, an additional 5 cases of HBV and 3 cases of HCV would also be diagnosed.

I read this review with great interest and asked myself the obvious question—surely BBV testing should replace HIV testing everywhere and always? However, in the *Lancet HIV* in 2015 I had already posed this question after leading the “Going Viral” combined BBV testing campaign a year earlier [9, 10]. “Going Viral” was the first-ever combined BBV testing week campaign. It was conducted across 9 emergency departments (EDs) in the United Kingdom. The campaign incorporated BBV testing into routine care to shed light on the prevalence of hepatitis B and C. The BBV prevalence was much higher than expected at 3.35%, 1.83% of which was HCV and 0.7% was HBV. These rates exceeded national prevalence estimates for hepatitis C and hepatitis B. Had only HIV alone been tested, as per national guidance, 54 viral hepatitis diagnoses would have been missed in the space of 1 week. As a result of our findings, we secured external funding to extend the testing period at the Royal London Hospital ED.

Over 9 months, we tested 6211 ED attendees and found that 4.1% had a BBV, mostly hepatitis C (2.4%). Half of those diagnosed with HBV required linkage to care [11]. A third of people who needed linkage to care had advanced disease, including 3 people with hepatocellular carcinoma. There were 5 BBV-related deaths. We also conducted interviews to understand whether by offering a test for BBVs rather than HIV alone, stigma in relation to HIV testing could be reduced [12]. Participant accounts indicated that the nontargeted approach of the combined BBV test seemed to be helpful. Routine opt-out BBV testing in the ED setting was viewed as an acceptable and valuable practice by the majority of patient and staff participants.

Nearly 10 years later, in January 2023, National Health Service (NHS) England reported on the achievements in the first 100 days of BBV testing between April and July 2022 [13]. A total of 250 000 HIV tests and >100 000 HCV antibody tests were delivered, resulting in identification of >500 people with previously unknown (or unrecognized or undiagnosed) BBVs. The report notes that opt-out testing in ED is an important mechanism to tackle health inequalities as it provides an inclusive approach to testing and tackling stigma associated with both HIV and viral hepatitis.

As a passionate early advocate for BBV testing, I remain surprised and disappointed that it took nearly 10 years until a cohesive national response occurred in England. Similarly, in Europe, while a combined BBV testing week took place in 2015, triple testing was not translated into routine clinical practice [14]. In contrast, in the United States, triple testing has been evaluated in an ED in Michigan as early as 2010 [15].

This strong argument made by Beard and Hill for the cost-effectiveness of BBV testing is clearly both necessary and timely. The authors acknowledge the practical barriers to implementing global testing, including adequate training of healthcare providers to deliver culturally appropriate testing to key populations. The authors highlight the central importance of combating stigma to increase uptake and acceptance of testing, regardless of test availability. They note that while $1 combination HIV/HBV/HCV triple tests are being developed, they are not yet approved by the WHO. Therefore, individual testing for HIV, HBV, and HCV is a low-cost interim solution that is only marginally costlier than HIV testing alone. In the future, this strategy also unlocks the future potential to include other infections with similar transmission routes such as syphilis.

The authors make the important points that universal access to these low-cost diagnostic tests *and* to antiviral treatment must be provided to avoid worsening health inequities. Curative treatment for HCV should follow a positive test and people with HIV and HBV should receive antivirals. Expanding diagnostic pathways is an opportunity that should be seized to expand preventive pathways—individuals who test negative for HBV should be offered vaccination. Individuals who test negative for HIV who would benefit from preexposure prophylaxis should receive it.

I join the authors in their recommendation that implementing BBV testing “will help to achieve the Sustainable Development Goal of elimination of these BBV epidemics by 2030.” We should not wait another moment to act on these important findings.

## Notes

***Potential conflicts of interest*.** C. O. has received research grants to her institution from Gilead Sciences, ViiV Healthcare, MSD, Janssen, and AstraZeneca and has received honoraria for advisory boards, lectureships, and travel scholarships from Gilead Sciences, ViiV Healthcare, MSD, and Janssen.

## References

[1] World Health Organization . Hepatitis B. 2023. Available at: https://www.who.int/news-room/fact-sheets/detail/hepatitis-b. Accessed 4 December 2023.

[2] World Health Organization . Hepatitis C. 2023. Available at: https://www.who.int/news-room/fact-sheets/detail/hepatitis-c. Accessed 4 December 2023.

[3] World Health Organization . World hepatitis day key messages. 2023. Available at: https://www.who.int/campaigns/world-hepatitis-day/2023/key-messages. Accessed 4 December 2023.

[4] Joint United Nations Programme on HIV/AIDS . Global HIV and AIDS statistics—act sheet. 2023. Available at: https://www.unaids.org/en/resources/fact-sheet. Accessed 4 July 2023.

[5] World Health Organization . SDG target 3.3—communicable diseases. Available at: https://www.who.int/data/gho/data/themes/topics/sdg-target-3_3-communicable-diseases. Accessed 4 December 2023.

[6] World Health Organization . Global health sector strategies on HIV, viral hepatitis and sexually transmitted infections for the period 2022–2030. 2022. Available at: https://www.who.int/publications-detail-redirect/9789240053779. Accessed 4 December 2023.

[7] Baumert TF , Berg T, Lim JK, Nelson DR. Status of direct-acting antiviral therapy for hepatitis C virus infection and remaining challenges. Gastroenterology 2019; 156:431–45. 30342035 10.1053/j.gastro.2018.10.024 PMC6446912

[8] World Health Organization . Consolidated guidelines on HIV, viral hepatitis and STI prevention, diagnosis, treatment and care for key populations. Geneva, Switzerland: World Health Organization; 2022. 36417550

[9] Orkin C , Wallis E. Should HIV testing week be blood-borne-virus testing week? Lancet HIV 2015; 2:e510–1. 26614964 10.1016/S2352-3018(15)00227-1

[10] Orkin C , Flanagan S, Wallis E, et al Incorporating HIV/hepatitis B virus/hepatitis C virus combined testing into routine blood tests in nine UK emergency departments: the “Going Viral” campaign. HIV Med 2016; 17:222–30. 26919291 10.1111/hiv.12364

[11] Parry S , Bundle N, Ullah S, et al Implementing routine blood-borne virus testing for HCV, HBV and HIV at a London emergency department—uncovering the iceberg? Epidemiol Infect 2018; 146:1026–35. 29661260 10.1017/S0950268818000870 PMC9184937

[12] Cullen L , Grenfell P, Rodger A, Orkin C, Mandal S, Rhodes T. ‘Just another vial…’: a qualitative study to explore the acceptability and feasibility of routine blood-borne virus testing in an emergency department setting in the UK. BMJ Open 2019; 9:e024085. 10.1136/bmjopen-2018-024085 PMC6501954 31048425

[13] NHS England . Emergency department opt out testing for HIV, hepatitis B and hepatitis C: the first 100 days. 2022. Available at: https://www.england.nhs.uk/long-read/emergency-department-opt-out-testing-for-hiv-hepatitis-b-and-hepatitis-c-the-first-100-days/#1-foreword. Accessed 4 December 2023.

[14] HIV in Europe . European HIV-hepatitis testing week 2015: results from the 2015 evaluation. 2015. Available at: https://www.testingweek.eu/media/2nbo0fv3/etw_2015-evaluation-report.pdf. Accessed 4 December 2023.

[15] Hall MR , Ray D, Payne JA. Prevalence of hepatitis C, hepatitis B, and human immunodeficiency virus in a Grand Rapids, Michigan emergency department. J Emerg Med 2010; 38:401–5. 18996668 10.1016/j.jemermed.2008.03.036

